# Antithrombotic therapy for venous thromboembolism in myeloproliferative neoplasms

**DOI:** 10.1038/s41408-018-0101-8

**Published:** 2018-06-26

**Authors:** Valerio De Stefano, Guido Finazzi, Tiziano Barbui

**Affiliations:** 1 Institute of Hematology, Catholic University, Roma, Italy; 2 IRCCS Policlinico Gemelli Foundation, Roma, Italy; 3 USC Hematology, ASST Papa Giovanni XXIII, Bergamo, Italy; 4 FROM Research Foundation, ASST Papa Giovanni XXIII, Bergamo, Italy

## Abstract

In myeloproliferative neoplasms (MPNs) the incidence of venous thromboembolism (VTE) is 0.6–1.0 per 100 pt-years, and the rate of recurrence after VTE is 6.0–6.5 per 100 pt-yrs. Vitamin K-antagonists (VKA) reduces the risk of recurrence after VTE at usual sites (i.e., deep venous thrombosis (DVT) of the legs and pulmonary embolism (PE)) by 48–69%, with a rate of recurrent thrombosis per 100 pt-yrs of 3.4–4.7 on VKA and 8.9–9.6 off VKA; VKA discontinuation produces a 2.2-fold increased risk of novel thrombotic events with respect to continuation. However, the rate of both recurrent thrombosis and major bleeding on VKA is higher in MPN patients than in non-MPN patients, and the risk-benefit balance of long-term VKA treatment is challenging. In the absence of strong evidence, the tailored management of MPN-related VTE should operatively consider the risk categories for recurrence and bleed well established in the non-MPN setting. In summary, MPN patients with VTE are candidates for life-long VKA treatment, especially after unprovoked proximal DVT and PE. Aspirin can offer a moderate benefit in those patients who stop anticoagulation. The use of direct oral anticoagulants should be explored aiming to ameliorate the rate of bleeding.

## Introduction

The updated classification of Philadelphia-negative myeloproliferative neoplasms (MPNs) includes polycythemia vera (PV), essential thrombocythemia (ET), prefibrotic/early primary myelofibrosis (prePMF), and primary myelofibrosis (PMF)^1^. The natural history of MPNs can be complicated by thrombotic complications or transformation into secondary myelofibrosis or leukemia^2^. The thrombotic burden is a target modifiable by medical intervention, and guidelines are aimed to develop effective strategies to this extent^3^.

Thromboses involve venous vessels in about one-third of cases. In contemporary cohorts of MPN patients the incidence of overall thrombosis/venous thromboembolism (VTE) per 100 patients-years was 2.6/1.0 in PV^4,5^, 1.9/0.6 in ET^6,7^, 1.9–2.1/0.6 in prePMF^6,8^, and 1.75/1.0 in PMF^9^. This incidence is definitely higher than that of the general population of Western countries, where the annual incidence of major VTE is between 0.1 and 0.2%^10^. In a recent population-based study, the rate of early VTE after diagnosis was nearly 10-fold increased in the MPN patients in comparison with the control participants, declining with the follow-up to a 3.2-fold increased rate^11^.

Although splanchnic venous thrombosis (SVT) and cerebral venous thrombosis (CVT) are greatly over-represented in comparison with the general population^12,13^, venous thrombosis at usual sites (i.e., deep venous thrombosis (DVT) of the legs with or without pulmonary embolism (PE), and isolated PE) have been reported to account for 54–77% of venous thrombotic events in PV^4,14^, 41% in ET^15^, and 89% in PMF^9^.

In PV venous thrombosis was associated with adverse prognosis contributing to the risk of death, but the weight of venous thrombosis at usual sites over SVT is not reported^4^. No specific data concerning the prognostic value of venous thrombosis in ET, prePMF or PMF are available.

Special attention is paid by experts to the treatment of MPN-related SVT, but no straight indication is given concerning acute DVT and PE in MPN^16,17^. Nevertheless, PE contributes to all the cardiovascular deaths for 8% in PV^18^, and for 33% in PMF^9^.

Therefore, in this paper, we will summarize recommendations for long-term management of VTE at usual sites in MPN patients, as derived from findings in either MPN and non-MPN patient cohorts.

### Risk factors for recurrent VTE

The American College of Chest Physicians (ACCP) guidelines for long-term treatment of VTE identifies as primary factors for estimating the risk of recurrence: the presence of a reversible provoking risk factor, unprovoked VTE, and presence of active cancer, which are the most important factors that influence the risk of recurrent VTE after stopping VKA^19,20^. Among patients with VTE provoked by a reversible factor, the risk of recurrence is much lower if the provoking factor was recent surgery compared with a nonsurgical trigger (eg, estrogen therapy, pregnancy, leg injury, a flight of >8 h). Additional factors considered strong enough to modify the duration of therapy after VTE are: isolated calf DVT vs. proximal DVT (relative risk, RR, approximately 0.5); one or more previous episodes of VTE (RR, approximately 1.5)^19,20^.

Other additional factors predicting the risk of recurrent VTE include: negative d-dimer 1 month after withdrawal of VKA (RR, approximately 0.4); presence of antiphospholipid antibodies (RR, approximately 2); inherited thrombophilia (RR, approximately 1.5); males vs females (RR, approximately 1.6); Asian ethnicity (RR, approximately 0.8); residual thrombosis in the proximal veins (RR, approximately 1.5)^19^.

Guidelines from the International Society of Thrombosis and Hemostasis (ISTH) suggest that the following factors may favor long-term anticoagulation in patients with a first unprovoked proximal DVT or PE: male gender; moderate-to-severe post-thrombotic syndrome; ongoing dyspnea (possibly related to unresolved or recurrent PE); satisfactory initial anticoagulant control; elevated D-dimer result based on individual D-dimer assay performance characteristics using a study-validated assay^21^.

In MPN patients, age older than 60 years and previous thrombosis are the major predictors of vascular complication^2,3^. In a multicenter series of 1545 contemporary PV patients, previous VTE (hazard ratio, HR, 2.6) and age > 65 years (HR 1.7) predicted venous thrombosis^4,5^. In a monocenter series of 587 PV patients, significant predictors of venous thrombosis were previous VTE, leukocytosis, and history of major bleeding^14^. In a study of 891 ET patients, only male gender predicted venous thrombosis (HR 2.0)^7^. In a multicenter analysis of 707 PMF patients, age > 60 years (HR 2.3) and the presence of the JAK2V617F mutation (HR 1.9) significantly predicted thrombosis, without difference between arterial and venous events^9^.

In patients with ET or PMF, three meta-analyses estimated an increased risk of thrombosis in JAK2V617F carriers in comparison with non-carriers. A higher association was reported for venous thrombosis (odds ratio, OR, 2.1 to 2.5) than arterial thrombosis (OR 1.7 to 2.0)^22–24^.

The frequency of inherited thrombophilic factors, such as factor V Leiden or prothrombin G20210A, was higher in MPN with venous thrombosis, similarly to the general population^25–27^. In ET the presence of both JAK2 V617F and inherited thrombophilia has been reported to produce an additive risk of thrombosis in younger patients^27^.

Summing up, in MPNs significant predictors of a first VTE are the previous history of VTE, older age, and inherited thrombophilia, similarly to the general population; moreover, the carriers of the JAK2 V617F mutation are more prone to venous thrombosis. The association with male sex and leukocytosis has been also reported.

In MPN patients who had an MPN-related thrombosis, a novel thrombosis preferentially involved the same arterial or venous district affected in the first event^28–30^. The risk factors for recurrence were age > 60 years^28^ and history of remote thrombosis;^29^ the subtype of MPN did not predict recurrence^28–30^.

### Treatment of VTE and duration of secondary prophylaxis

#### Acute treatment with heparin

DVT of legs or PE in MPN patients should be treated the same as DVT or PE occurring in the non-MPN patients^19^. Therefore, low-molecular-weight heparin (LMWH) or fondaparinux is suggested over i.v. or s.c unfractionated heparin; early initiation of vitamin K-antagonists (VKA) aiming for targetting an international normalized ratio 2.5 (range 2.0–3.0) is recommended^19^. At present, there are no data to support in MPN patients with VTE the current ACCP recommendation of early use of direct oral anticoagulants (DOACs) in non-cancer patients with VTE^20^.

Relatively frequent cases of heparin-induced thrombocytopenia have been reported in MPN patients so that special care is due during the heparin course in monitoring a drop of the platelet count^31–33^.

#### The rate of recurrent thrombosis and secondary antithrombotic prophylaxis

After the first episode of VTE, the duration of secondary prophylaxis with VKA should be decided to balance the risk of hemorrhagic complications with that of a novel VTE. In the general population the cumulative rate of recurrence after discontinuation of anticoagulation is as high as 39.9% within 10 years from the first VTE^34^, being lower in patients with VTE associated with transient risk factors and maximal in those with unprovoked VTE^34–36^.

In the MPN patients, the rate of recurrent thrombosis and the effects of secondary prophylactic treatments have been specifically addressed by three studies^28–30^. In an Italian retrospective cohort of 494 patients with PV (*n* = 235) or ET (*n* = 259) and with a previous thrombosis, 114 had VTE at usual sites (i.e., DVT of the legs and/or PE) and 42 had VTE at unusual sites (i.e., SVT and CVT). Thrombosis recurred in 166 patients (33.6%), with an incidence of 7.6 per 100 pt-years^28^. In a retrospective Spanish cohort of 150 MPN patients receiving VKA after a first thrombosis, 75 of them had DVT of the legs and/or PE. The overall rate of recurrent thrombosis was 6.0 per 100 pt-years^29^. In a recent multicenter international retrospective survey, a homogeneous cohort of 206 MPN patients with DVT of the legs and/or PE were recruited, 155 of them receiving VKA after the index event. The overall rate of recurrent thrombosis per 100 patient-years was 6.5^30^.

#### Vitamin k-antagonists

In the Italian cohort from the Gruppo Italiano Malattie Ematologiche dell’Adulto (GIMEMA) multivariable analysis showed that VKA effectively prevented recurrence in patients with VTE (hazard ratio, HR, 0.32, 95% CI 0.15–0.64). After exclusion of the patients with VTE at unusual sites, in the remaining 114 patients, long-term treatment with VKA remained effective at preventing recurrence (HR 0.31, 95%CI 0.13–0.69)^28^.

In the Spanish cohort from the Grupo Español de Enfermedades Mieloproliferativas Filadelfia Negativas (GEMFIN) the rate of recurrent thrombosis per 100 pt-years was 4.5 on VKA and 12.0 off VKA, *p* < 0.0005; in the patients with DVT and/or PE, the rate of recurrent VTE was 3.4 per 100 pt-years on VKA and 9.4 per 100 pt-years off VKA (*p* = 0.016)^29^.

In the international cohort recruited from the European LeukemiaNet (ELN) the rate of recurrent thrombosis per 100 patient-years was 4.7 on VKA and 8.9 off VKA (p = 0.03); consistently, the rate of recurrent VTE per 100 patient-years was 3.7 on VKA and 7.1 off VKA (*p* = 0.04). A sensitivity analysis by treatment showed that the rate of recurrent VTE per 100 patient-years was 4.2 among the patients who continued VKA and 9.6 after discontinuation of VKA (*p* = 0.03), with a 2.2-fold increased risk of novel thrombotic events after discontinuation^30^.

Interestingly, an indirect comparison of this cohort of MPN patients with VTE with non-MPN patients with VTE recruited in recent trials and receiving VKA suggests a higher thrombotic potential in MPN patients. In fact, in this study, the cumulative probability of recurrent thrombosis at 1 year of VKA treatment is 7.8%^30^, definitely higher than the probabilities of recurrent thrombosis on VKA reported in the recent trials, which were between 1.8 and 3.5%^37–40^. Accordingly, the cumulative probability of recurrent thrombosis after discontinuation of VKA was 42% at 5 years from the withdrawal of treatment^30^, which was higher than the 29.1% rate observed at 5 years in the non-MPN patients^34^.

#### Low-molecular-weight heparin

Cancer patients with VTE are at increased risk for both bleeding and VTE recurrence^41^. Based largely on the CLOT and CATCH trials^42,43^, current guidelines recommend LMWH for at least 3–6 months in cancer-associated VTE, suggesting to treat indefinitely patients with active malignancy and ongoing treatment, on the basis of the superior safety and efficacy compared to VKA^19,20^. Given the neoplastic nature of MPN, such an approach could be attractive also in these patients. However, there are several considerations that should be kept in mind. First, at variance with most cancers, the MPN disease activity persist for decades, so that continued life-long treatment with daily s.c. heparin can be troublesome. Second, in MPN there is suspicion of an increased risk of heparin-induced thrombocytopenia^31–33^, so that caution should be adopted in prescribing long-term treatment. Finally, published evidence about efficacy and safety of long-term administration of LMWH in MPN is not enough to establish recommendations for clinical practice; in the ELN cohort of 206 MPN patients with VTE, 9.2% received extended LMWH prophylaxis after the first VTE event, but the duration was between 6 and 12 months only in four of them (De Stefano et al, unpublished data)^30^.

#### Aspirin

In the GIMEMA cohort, multivariable analysis showed that besides VKA, also antiplatelet agents effectively prevented recurrence in patients with VTE (HR 0.42, 95% CI 0.22–0.77). However, in the 114 patients with VTE at usual sites, long-term treatment with aspirin did not retain statistical significance in preventing recurrence (HR 0.53, 95%CI 0.27–1.03)^28^.

#### Cytoreductive treatment

Cytoreductive treatment (hydroxyurea in 82% of the cases) halved the risk in the overall GIMEMA cohort (HR 0.53; 95% CI 0.38–0.73); however, a secondary analysis confirmed the efficacy of cytoreductive treatment only in patients with first arterial thrombosis (53% reduction in the risk), whereas in patients with first VTE the influence was more limited (34% reduction in the risk) and not statistically significant^28^.

In the ELN cohort, the patients receiving only VKA after VTE in the absence of cytoreduction showed no significant difference in the incidence rate of recurrent thrombosis per 100 patient-years observed in patients receiving both VKA and cytoreduction (6.3, 95% CI 1.3–18.6 and 4.4, 95% CI 2.5–7.2, respectively, *p* = 0.57)^30^.

#### The rate of major bleeding during antithrombotic treatment

In the GEMFIN cohort, there was no significant difference in hemorrhagic events based on whether the patients were on VKA or not on VKA (1.8 vs. 1.5 per 100 pt-years, respectively)^29^.

In the ELN cohort, the incidence of major bleeding per 100 patient-years was 2.4 on VKA and 0.7 off VKA^30^. The cumulative probability of major bleeding at 1 year of VKA treatment was 2.8%^30^, which appears higher than the counterpart value of 1.2–2.2% recorded in the VKA arms of trials comparing VKA vs. DOACs^37–40^.

In the GIMEMA cohort, the association of antiplatelet agents plus VKA increased major bleedings per 100 pt-years compared with antiplatelet agents or VKA alone (2.8 vs. 0.8 and 0.9, respectively)^28^.

### Perspective on the use of direct oral anticoagulants in MPNs

DOACs dabigatran, rivaroxaban, apixaban, and edoxaban are approved for the treatment of acute VTE^20^. Subgroup analyses of these patients, either pooled or separately reported, suggest that DOACs could be an efficacious alternative to VKA therapy for the treatment of cancer-associated VTE. In the Hokusai-VTE trial, edoxaban was found non-inferior to warfarin for the treatment of cancer patients with VTE with respect to recurrence (4 vs. 7%); major bleeding occurred in 3% of the patients who received either edoxaban or warfarin^44^. In one pooled analysis, 514 patients with active cancer who were treated with a DOAC were compared to 459 treated with a VKA. The pooled incidence rate of recurrent VTE during DOAC therapy was 4.1% compared to 6.1% with VKA (HR 0.66, 95% CI 0.38–1.2). The rate of major and clinically relevant non-major bleeding was similar in both groups (15 vs 16%, RR 0.94, 95% CI 0.7–1.3)^45^.

On the other hand, in the Hokusai VTE Cancer trial, cancer patients with VTE were randomized to edoxaban or s.c. dalteparin. The rate of recurrent VTE was lower (7.9 vs. 11.3%) but the rate of major bleeding was higher with edoxaban than with dalteparin (6.9 vs. 4.0%). This difference was mainly due to the higher rate of upper gastrointestinal bleeding with edoxaban in patients with gastrointestinal cancer^46^.

MPN patients are prone to either thrombotic and hemorrhagic increased risk, and in this setting, the use of DOACs could ameliorate the bleeding risk with respect to the use of VKA^47^. However, the published experience is very limited and does not allow any conclusion. In the Hokusai trials the patients with hematological malignancies recruited in the different arms (edoxaban, VKA, LMWH) were approximately 10%, mostly with leukemia and lymphoma^44,46^, so that any result is difficult to be generalized for MPN patients. In the aforementioned ELN cohort of MPN patients with DVT of the legs and/or PE only 3.3% of patients were treated with DOACs^30^. In a single-center registry of 760 MPN patients, 25 (3.3%) were treated with a DOAC. The reasons for prescribing DOACs were atrial fibrillation and thrombotic events for 13 and 12 patients, respectively. Only 8 of them received DOACs because of VTE^48^. In the German MPN registry of the Study Alliance Leukemia, 68 out of 454 patients (14.9%) had suffered from DVT or SVT; only 8 patients (1.7% of the cohort) were treated with rivaroxaban. However, multivariate analysis revealed an odds ratio of major bleeding for patients on rivaroxaban of 1.61 (non-significant), which is lower than those for patients on VKA (1.97), double platelet inhibition (3.05) and heparin (5.64, the only drug with a significant odds ratio for major bleeding)^49^.

## Conclusions

At present, there is not a clear decision guide for the optimal duration of anticoagulant treatment after an MPN-related VTE, and the lack of firm evidence in this setting is mirrored by a recent survey that showed a marked heterogeneity in the opinion of hematologists regarding this issue^50^.

**Fig. 1:**
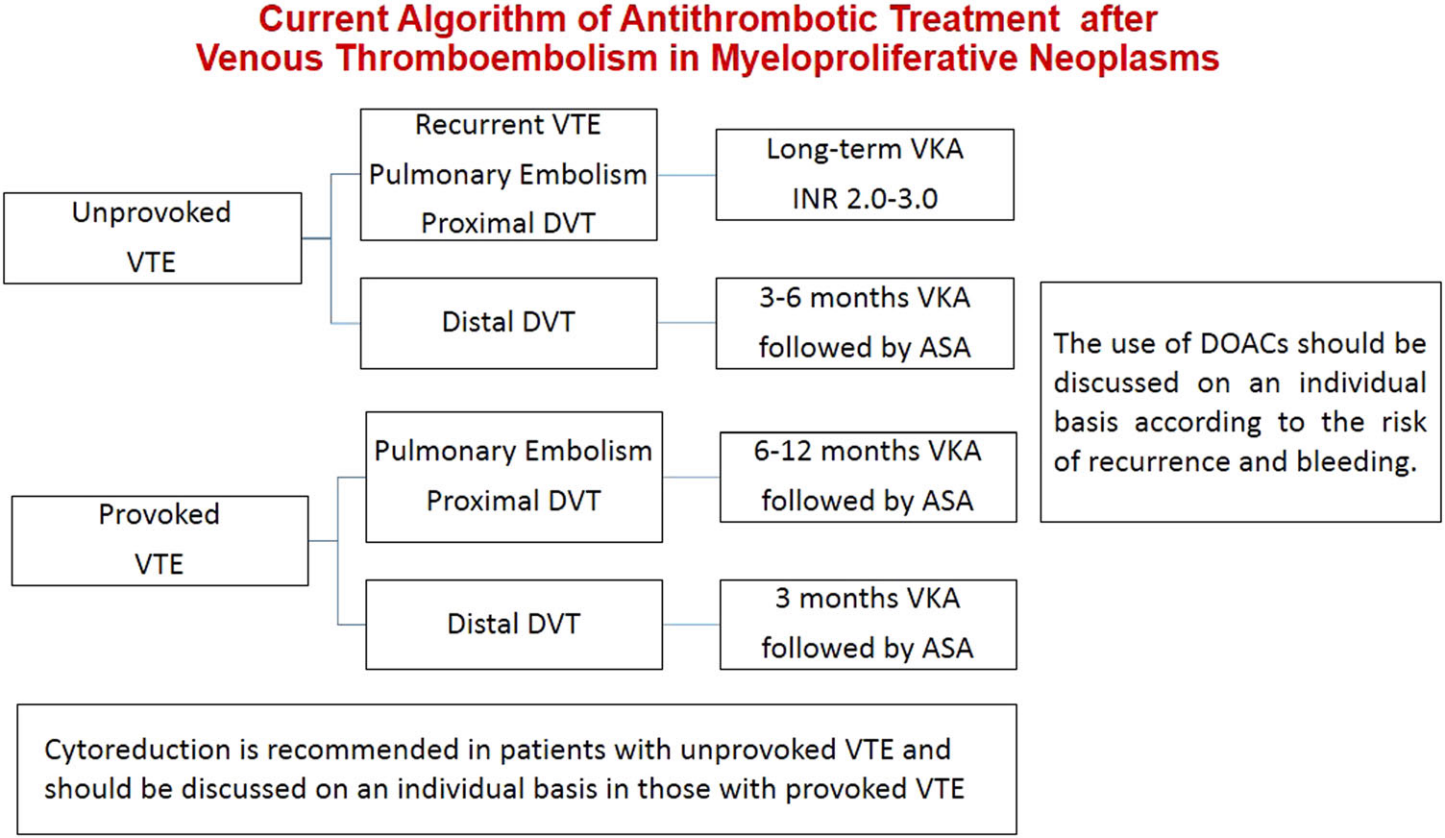
Current treatment algorithm in venous thromboembolism at usual sites in myeloproliferative neoplasms

In MPN patients, the overall rate of recurrent thrombosis after VTE is 6.0 to 6.5 per 100 patient-years;^29,30^ long-term treatment with VKA (INR 2.0–3.0) is associated with a clear benefit, reducing the incidence rate of recurrence from 48 to 69% with respect to off-treatment^28–30^. Accordingly, discontinuation of VKA treatment produces in patients off therapy a 2.2-fold increased risk of novel thrombotic events in comparison with patients who continued treatment^30^. In non-MPN patients, anticoagulation with VKA or DOACs is recommended, suggesting an indefinite duration for patients with unprovoked first VTE or for those patients with permanent risk factors, such as cancer^19,20^. Some decisional criteria such as the circumstances of the first event (i.e., unprovoked or provoked) and the location of DVT (i.e., proximal or distal) are widely employed in the non-MPN patients to plan the duration of oral anticoagulation; additional criteria such as pulmonary embolization, male sex, D-dimer level, residual vein thrombosis, thrombophilia can refine and score the risk profile. In MPN patients with VTE those putative criteria for recurrence have not been validated, likely due to the weight of major risk factors such as MPN diagnosis itself which could obscure the role of other predictors. However, well established and strong risk factors such as unprovoked previous VTE or proximal DVT or recurrent VTE can be operatively applied in tailoring decisions for MPN patients (Fig. 1). There is evidence either in non-MPN patients^20^ and in MPN patients^28^ with VTE that aspirin can offer a moderate protection from recurrence after discontinuation of VKA, so that can be considered in patients who are stopping anticoagulant therapy and do not have a contraindication to aspirin, as suggested by the ACCP guidelines (Fig. 1)^20^.

Cytoreduction reduces significantly and independently the risk of recurrence in MPN patients having suffered from arterial thrombosis^28^ or ischemic stroke^51^, whereas in patients with first VTE the reduction in the risk of recurrence was weaker and not statistically significant^28,30^. Finally, each therapeutic decision must face the higher potential for bleeding present in MPN patents with respect to the anticoagulated non-MPN patients. In particular, patients with a history of major bleeding are paradoxically prone both to venous thrombosis^14^ and to novel bleeding during VKA^20^ so that this special population challenges for new prophylactic strategies. Other risk factors for bleeding are age > 75 years, comorbidity, poor INR control^20^. The use of DOACs is associated with a lower rate of bleeding so that the potential benefit in those patients at high-risk of re-thrombosis and major bleeding should be urgently investigated. In the meanwhile, we suggest that patients with VTE at higher bleeding risk and candidates to long-term oral anticoagulation should be considered for treatment with DOACs instead of VKA (Fig. 1).

Recurrent thrombosis can occur in MPN patients on VKA treatment with INR in the therapeutic range; in the patients of the GIMEMA cohort with INR value available at the time of recurrence 50% (6 of 12) had INR within the therapeutic range^28^. There is no published evidence about to tackle this circumstance in MPN patients; as suggested by the ACCP guidelines for the non-MPN patients, a temporary switch to LMWH for at least 1 month can be considered^20^. After the acute phase, efforts aimed to obtain an INR target more intense than 3.0 or switching to a combined treatment of VKA plus aspirin should be considered not evidence-based and potentially harmful due to the increased bleeding risk.

In conclusion, in the MPN patients, the secondary antithrombotic prophylaxis after VTE is unsatisfactory because of suboptimal efficacy and safety. Multicenter retrospective and prospective trials are needed to improve treatment strategies.
